# Novel Solventless Extraction Technique to Preserve Cannabinoid and Terpenoid Profiles of Fresh Cannabis Inflorescence

**DOI:** 10.3390/molecules26185496

**Published:** 2021-09-10

**Authors:** Ethan B. Russo, Jeremy Plumb, Venetia L. Whiteley

**Affiliations:** 1 CReDO Science, Austin, TX 78753, USA; nw@credo-science.com; 2 Production Science, Prūf Cultivar, Portland, OR 97211, USA; contact@jeremyplumb.com

**Keywords:** cannabis, cannabinoid, terpene, terpenoid, essential oil, botanical medicine, pharmaceutical, extraction, hemp, hashish

## Abstract

Despite its use by humans for thousands of years, the technology of cannabis usage and extraction is still evolving. Given that the primary pharmacological compounds of interest are cannabinoid and terpenoids found in greatest abundance in capitate glandular trichomes of unfertilized female inflorescences, it is surprising that older techniques of hashish making have received less technological advancement. The purpose of this study was to employ organically grown cannabis and to isolate pure trichomes from freshly picked flowers via exposure to vapor from solid CO_2_, commonly known as “dry ice”, followed by their isolation via sifting through a 150 µ screens while maintaining the cold chain. Biochemical analysis was undertaken on fresh flower, frozen-sifted flower by-products, treated trichomes (Kryo-Kief™), dried flower, dried sifted flower by-product and dried kief. The dry ice process successfully concentrated cannabinoid content as high as 60.7%, with corresponding concentration and preservation of monoterpenoids encountered in fresh flower that are usually lost during the conventional cannabis drying and curing process. The resulting dried sifted flower by-product after dry ice processing remains a usable commodity. This approach may be of interest to pharmaceutical companies and supplement producers pursuing cannabis-based medicine development with an eye toward full synergy of ingredients harnessing the entourage effect.

## 1. Introduction

Phytocannabinoids and cannabis terpenoids are the primary medicinal components for most cannabis-based medicines. Heretofore, cannabis extraction and processing has generally operated under the assumption that the material would be smoked. In modern times, this approach of drying and curing is outmoded and counterproductive, adding unnecessary steps, costs and reducing monoterpenoid content while retaining extraneous components. Phytocannabinoids and cannabis terpenoids are produced in greatest abundance in the capitate glandular trichomes of unfertilized female inflorescences. Given the volume of a sphere as 4/3πr^3^, a 150 µ diameter capitate glandular trichome on the bract of a cannabis inflorescence would have a volume of 1.77 × 10^−3^ mm^3^ as compared to a 30 µ diameter sessile trichome on a leaf with a volume of 1.41 × 10^−5^ mm^3^, which is a two order of magnitude difference. Additionally, sessile trichomes are qualitatively different biochemically, favoring bitter sesquiterpenes over monoterpenes to dissuade predatory grazing [1,2,3]. As such, cannabis flowers may contain phytocannabinoid concentrations 18–20 fold greater than the leaves [1].

Beyond the trichomes, most of the other biomass materials in the flowers, leaves and other plant parts are actually extraneous to the majority of cannabis medicine preparations. Their inclusion in extraction may be counterproductive via the inclusion of chlorophyll, lipid components and many other pharmacologically unnecessary compounds. Phytocannabinoids and terpenoids are secreted into and contained within the trichome envelope. Properly speaking, these contents are the key “active pharmaceutical ingredients” (APIs) of most medicinal cannabis preparations.

Cannabis is commonly dried and cured prior to use for months in the sun as in the form of hashish produced in the Rif mountains of Morocco, variously rendered as *kif*, *kief* or *keef* in English [4,5]. This process oxidizes some tetrahydrocannabinol (THC) to cannabinol (CBN), and it actually converts myrcene into a distinct rare terpene, 5,5-dimethyl-1-vinylbicyclol[2.1.1]hexane (dubbed “hashishene”) [6]. Alternatively, cannabis may be dried and cured under controlled humidity conditions, as in the preparation of the cannabis-based pharmaceuticals, nabiximols and cannabidiol such as Epidiolex^®^ (GW Pharmaceuticals, UK) [1,7]. The original intent of this drying process was to improve “smoke-ability” by oxidation of chlorophyll to the diterpene phytol [2] and to reduce chances of mold formation. In the process, the “headspace volatiles” of cannabis, which are the lower molecular weight monoterpenoids, are lost during drying and “curing”, ranging from 31 to 55.2% when dried at room temperature, depending on the length of the process [8].

If one assumes that the entourage effect of synergy between phytocannabinoid and terpenoid components is a valid concept in cannabis therapeutics [2,3,9,10] (vide infra), this will militate the need for novel processes to preserve the biochemical profile of fresh flowers. This investigation will focus on a new approach for mitigating these problems: a novel solventless extraction technique (Kryo-Kief™; patent pending) that preserves the cannabinoid and terpenoid profiles of the fresh flower. Additional approaches for preserving monoterpenoid content in secondary extraction will follow (*vide infra*, the Discussion section).

The extraction technique described here is designed to isolate the largest capitate glandular trichomes from freshly harvested cannabis inflorescences. This is performed in order to preserve the native biochemical profile of phytocannabinoids and terpenoids, including the headspace volatile monoterpenoids that are generally lost in other processes. This is achieved by a process of exposure to vapor from dry ice (solid CO_2,_ surface temperature of −78.5 °C) to produce a lyophilization effect as water content is sublimated and material dehumidified. This is then followed by a sifting process in a maintained cold chain in order to isolate trichomes (*vide infra*, Materials and Methods). Analysis was performed on each of six conditions for four cannabis chemovars: fresh flower, frozen-sifted flower by-product, Kryo-Kief™ dry ice process, dried flower, dried sifted flower and dried kief. This process demonstrated the highest concentrations of cannabinoids and terpenoids in the Kryo-Kief™, with preservation of profiles of fresh flower.

## 2. Results

### 2.1. Analysis of Treatment Pilot of Doug Fir Type I Chemovar

**Figure 1:**
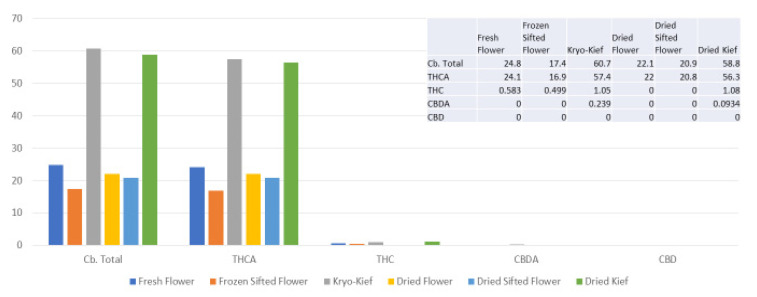
Doug Fir Type I chemovar cannabinoid assay results (all figures are %).

The amount of 100 g of fresh cannabis flower was processed for only one hour of dry ice vapor exposure and five minutes of Pollinator sifting treatment (*vide infra*, Methods, Section 4.9). Despite this, the Kryo-Kief™ dry ice process produced the highest cannabinoid yields, concentrating the total from 24.8 to 60.7% (Extraction ratio: 2.5×) and tetrahydrocannabinolic acid (THCA) from 24.1 to 57.7% (Extraction ratio: 2.4×) (Figure 1). For these calculations, the following equation was utilized.

Percentage of component in concentrate ÷Percentage of component in fresh flower=Extraction ratio

**Figure 2:**
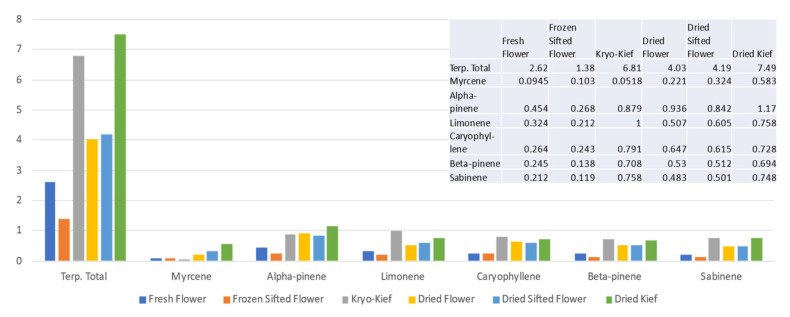
Doug Fir Type I chemovar terpenoid assay results (all figures are %).

Terpenoid analysis of this sample showed concentrations of the total from 2.62 to 6.81% (Extraction ratio: 2.6×) and limonene from 0.324 to 1% (Extraction ratio: 3.1×) (Figure 2).

**Figure 3:**
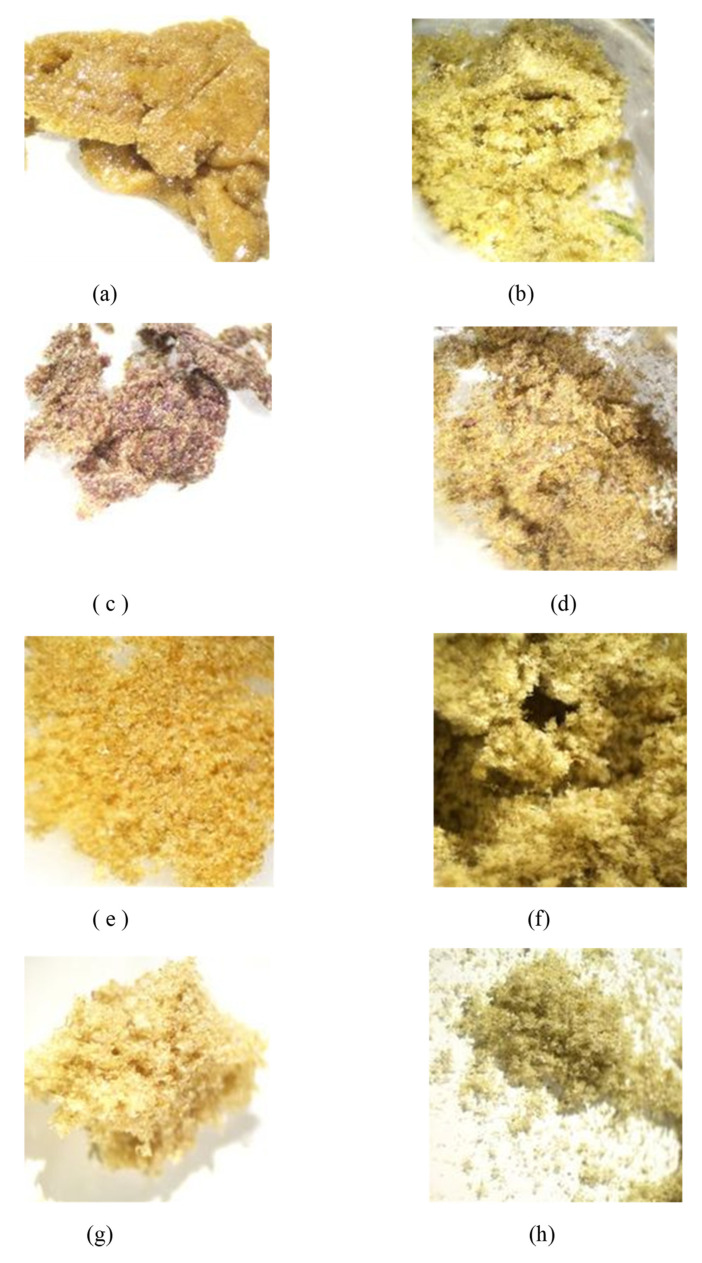
(**a**) Sample of Kryo-Kief™ from Doug Fir Type I chemovar; (**b**) sample of dried kief from Doug Fir Type I chemovar; (**c**) sample of Kryo-Kief™ from Astral Works Type II chemovar; (**d**) sample of dried kief from Astral Works Type II chemovar; (**e**) sample of Kryo-Kief™ from Tangie Biscotti Type I chemovar; (**f**) sample of dried kief from Tangie Biscotti Type I chemovar; (**g**) sample of Kryo-Kief™ from Ursa Major Type I chemovar; (**h**) sample of dried kief from Ursa Major Type I chemovar (images provided with permission of Lightscale Labs, Portland, OR, USA).

Dry ice kief production from the shortened treatment was low, compounded by this first sample sticking to porous paper. Only 0.5 g was collected, representing 0.5% of original wet weight. The dried kief, in contrast, contained leaf fragments and other extraneous particulate material with a yield of 0.28% of original wet weight (Figure 3). For these calculations, the following equation was utilized.

Weight of fresh flower ÷Weight of kief=Percentage of original wet weight.

### 2.2. Analysis of Treatment Pilot of Astral Works Type II Chemovar

**Figure 4:**
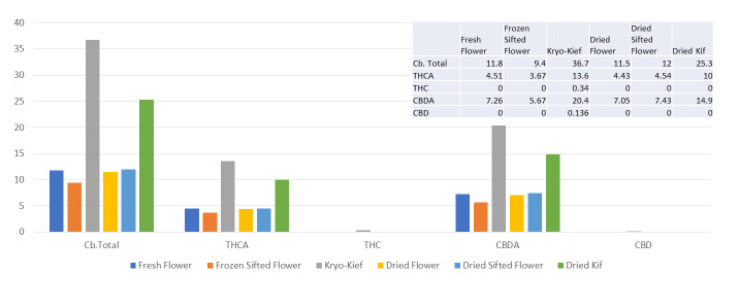
Astral Works Type II chemovar cannabinoid assay results (all figures are %).

Once more, 100 g of fresh flower was processed for only one hour of dry ice vapor exposure and five minutes of Pollinator treatment (*vide infra*, Methods, Section 4.9). In this trial, cannabinoid yields of dry ice kief were notably greater than for other samples, increasing the total from 11.8 to 36.7% (Extraction ratio: 3.1×), with THCA increasing from 4.51 to 13.6% (Extraction ratio: 3×) and cannabidiolic acid (CBDA) from 7.26 to 20.4% (Extraction ratio: 2.8×) (Figure 4).

**Figure 5:**
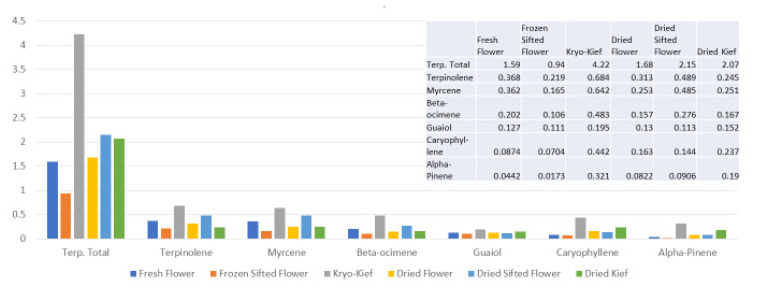
Astral Works Type II chemovar terpenoid assay results (all figures are %).

The terpenoid analysis demonstrates much higher yields for dry ice kief in total, from 1.59 to 4.22% (Extraction ratio: (2.7×)) and for each specific compound (Figure 5).

The Kryo-Kief™ yield was 0.1 g or 0.1% of original fresh wet weight (Figure 3c), compared to 0.08 g or 0.08% of wet weight for the dried kief (Figure 3d). Anthocyanin pigmentation, which is a desirable market trait, is maintained in dry ice kief.

### 2.3. Analysis of Treatment of Tangie Biscotti Type I Chemovar

**Figure 6:**
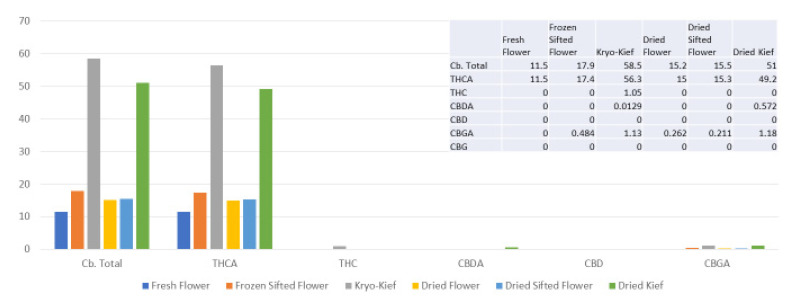
Tangie Biscotti Type I chemovar cannabinoid assay results (all figures are %).

This sample was run with 200 g of fresh flower with an extended 48 h of dry ice vapor exposure and 20 min of Pollinator treatment (Methods, Section 4.9). Once more, cannabinoid yields were the highest for dry ice kief, with the total increasing from 11.5 to 58.5% (Extraction ratio: 5.1×) and THCA from 11.5 to 56.3% (Extraction ratio: 4.9×) (Figure 6).

**Figure 7:**
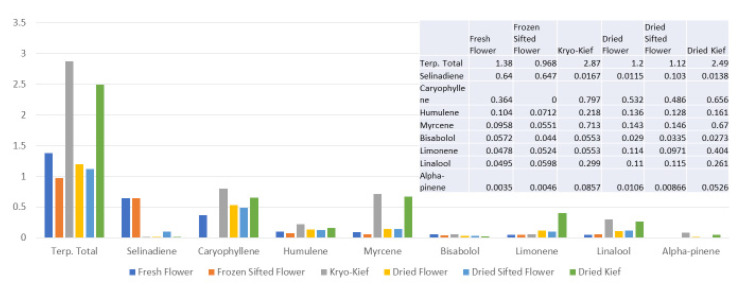
Tangie Biscotti Type I chemovar terpenoid assay results (all figures are %).

Terpenoid total increased from 1.38 to 2.87% (Extraction ratio: 2.1×), with most individual compounds highest in dry ice kief, notably linalool concentrating from 0.0495 to 0.299% (Extraction ratio: 6×) (Figure 7). The dry ice kief appears extremely clean and lighter with rare green flecks (Figure 3e), with a yield of 8.12 g or 4.06% of the original wet flower weight as compared to dried kief with many more particulates and yield of 1.68 g or only 0.84% of fresh weight (Figure 3f).

### 2.4. Analysis of Treatment of Ursa Major Type I Chemovar

**Figure 8:**
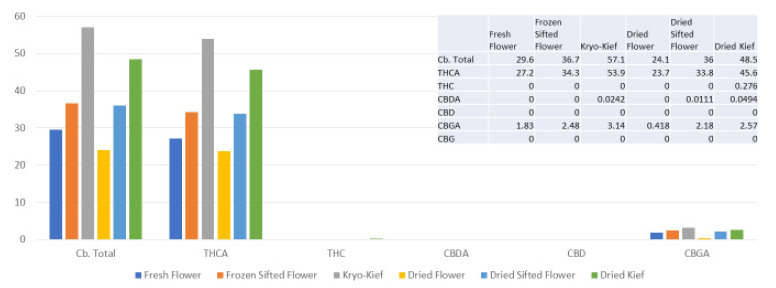
Ursa Major Type I chemovar cannabinoid assay results (all figures are %).

**Figure 9:**
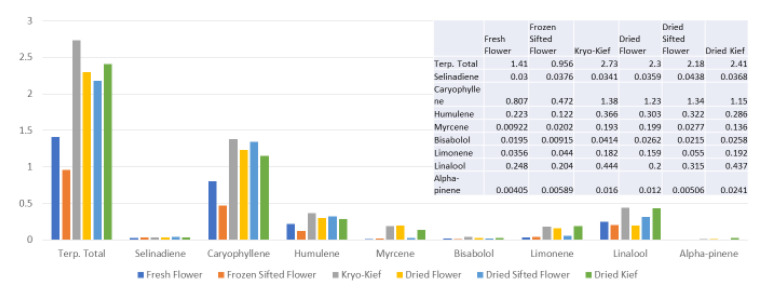
Ursa Major Type I chemovar terpenoid assay results (all figures are %).

This sample was also treated with dry ice vapor for an extended 48 h and 20 min of pollinator extraction. Cannabinoid total increased from 29.6 to 57.1% (Extraction ratio: 1.9×), with THCA from 27.2 to 53.9% (Extraction ratio: (2×)) and cannabigerolic acid (CBGA) from 1.83 to 3.14% (Extraction ratio: 1.7×) (Figure 8). Terpenoid total increased from 1.41 to 2.73% (Extraction ratio: 1.9×) and linalool from 0.248 to 0.444% (Extraction ratio: 1.8×) (Figure 9). The dry ice kief from this sample is notably cleaner with no green chlorophyll tinge (Figure 3g). A Kryo-Kief™ yield of 6.96% of original fresh weight was ultimately achieved with this chemovar than compared to 1.33% for dried kief (Figure 3h). 

## 3. Discussion

The current investigation has demonstrated a practical solventless method for concentrating phytocannabinoids and terpenoids as they appear in fresh inflorescences, thus providing less complex base materials for subsequent usage with the ability to maintain the acid cannabinoids or with the option to pursue subsequent decarboxylation to neutral cannabinoids. The degree of concentration of phytocannabinoids produced here is noteworthy in comparison to traditional sieving as reported in past research on hashish production with yield of 40% THC from the chemovar “Skunk #1” or a 50–55% THC putative upper limit yield with high-tech sieving [11]. Similarly, the achieved concentrations are similar to those realized in solvent extractions (vide infra).

The preservation of lower molecular weight monoterpenoids demonstrated is of particular interest in comparison to prior studies that document significant wastage with drying and curing [8]. Such findings have been corroborated in a more recent study in which fresh varieties expressed higher monoterpene content while dried samples demonstrated lower concentrations after a loss of the lower molecular weight compounds with lower boiling points [12]. While the differences between fresh and dried preparation terpenoid concentrations observed here are less prominent than in the study of Ross et al. [8], this can be attributed to the advanced drying technology regimen applied herein, employing low ambient temperature (vide infra, Methods Section 4.6).

The question remains whether such efforts to harness potential synergy of the cannabis entourage are necessary or desirable for the cannabis consumer, irrespective of whether such use be medical or adult. Two recent studies have demonstrated a lack of interaction among common terpenoids on CB_1_ receptors [13,14] but without accounting for other mechanisms of action that might produce such synergy, whether by boosting therapeutic effects or reducing adverse events attributable to THC [2]. A recent study did show cannabimimetic effects by common cannabis terpenoids [15] but at concentrations at or above 100 µM. It is highly likely that consumers distinguish differences in the subjective experience that they experience from different cannabis chemovars [9]. This, in turn, may account for the thousands of varieties available in legal and clandestine markets. Contemporaneously, some objective evidence for corroboration of such synergistic entourage effects is emerging [9]. One example is the preliminary finding that limonene reduced anxiety from THC inhalation in a dose-responsive manner in humans in a randomized controlled trial setting [16]. Addition experiments underway may further elucidate phytocannabinoid/terpenoid interactions in randomized controlled trials.

**Figure 10:**
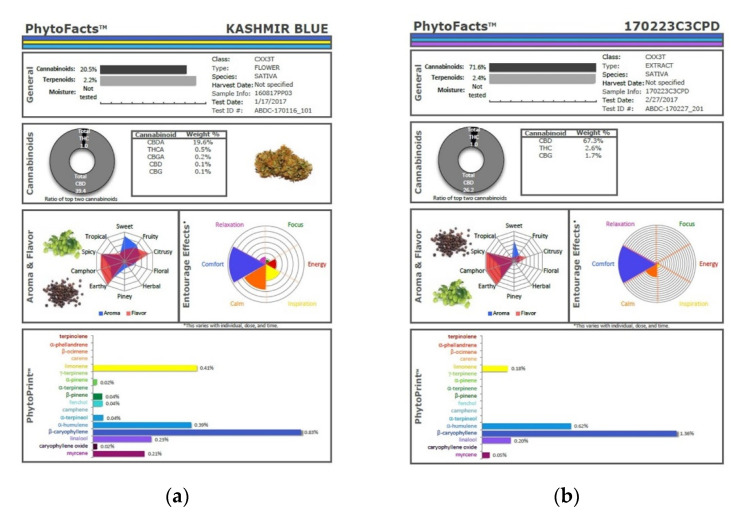
Comparison of cannabinoid and terpenoid profiles from (**a**) Kashmir Blue Type III chemovar flower; (**b**) corresponding cannabinoid and terpenoid profiles after conventional “one pass” supercritical CO_2_ extraction.

The technique presented here may represent an advantage in that various secondary extraction techniques can exacerbate the problem of monoterpenoid wastage. Supercritical CO_2_ extraction has been very attractive for its efficiency in preserving cannabinoids and its avoidance of solvent residues, but as it is normally performed in a single pass, this technique yields markedly reduced monoterpenoid fractions in favor of sesquiterpenoids, as illustrated (Figure 10), in which phytocannabinoid concentration is increased from 20.5 to 71.6% (Extraction ratio: 3.5×) from flower to extract, while terpenoid total rises only from 2.2 to 2.4% (Extraction ratio: 1.1×) and with wastage of limonene in particular. This is in marked contrast to the preservation and concentration of limonene in the dry ice kief process (Figure 7 and Figure 9). Given the ability of limonene to reduce THC-associated anxiety and to elevate mood [2,16], a loss of clinical efficacy is certainly possible. Similarly, myrcene concentrations reduced by supercritical CO_2_ extraction (Figure 10) are enhanced in dry ice kief. Myrcene is noted to have anti-inflammatory and sedative effects that may be clinically valuable in various conditions [2].

Monoterpenoid losses have been similarly documented [17] in a study in which a supercritical CO_2_ extracted concentrated cannabinoids and sesquiterpenoids while reducing the final values for monoterpenoids, with the conclusion that such products have significantly altered biochemical profiles than their flower source with different fragrances and flavors as well.

Such problems of CO_2_ extraction may be avoided by eliminating the temptation to accomplish the task in one procedure; rather, separate dedicated passes for phytocannabinoid and terpenoid fractions may be preferable, with a subsequent mixture of the two runs. Another alternative was demonstrated with focused ultrasound extraction (FUSE) in which optimal conditions for extraction were ascertained to be 100 bar, 35 °C and 1 mL/min with no co-solvent for terpenes and with 20% ethanol as co-solvent for cannabinoids [18]. FUSE was more efficient than supercritical fluid extraction, with an 80% yield of essential oils (EOs) and cannabinoids (CBs) in the first pass.

Cold ethanol provides an alternative that actually is more efficient than CO_2_ in terpenoid extraction. Simple filtration of the resulting material obviates the need for a separate “winterization” step in order to remove the waxy cell wall ballast from flowers (that adversely affects taste and impairs shelf-life) [1]. Tinctures result directly, or the material may alternatively be roto-vaped to purge ethanol for concentrate production and vaporization, etc.

Steam distillation is the primary method of producing essential oils (terpenoid fraction), sometimes without significant cannabinoid extraction. When derived from cannabis, these materials could be utilized as the source material to “improve” cannabis extracts to conform to desired specifications [9]. In the New York state cannabis program, it is mandatory that terpenoids in all cannabis products must be cannabis-derived.

A remaining issue for dry ice kief would be decarboxylation of the native acid cannabinoids to their neutral counterparts (THC, CBD and cannabigerol (CBG), etc.). Soxhlet extraction employs a closed system with heating that can achieve decarboxylation of the base material without terpenoid evaporation, but with the possibility of some bioconversion to other forms.

Kryo-Kief™ may represent a “value-added” proposition in that the process yields two end products: the dry ice kief itself and the remaining frozen-sifted flower by-product. As is evident from the analyses in the above figures, the latter still retains significant phytocannabinoid and terpenoid concentrations in material that is not tainted by water (as in water hash extraction) nor solvents. Thus, it may be useful feedstock for subsequent secondary extraction via other techniques. In other words, nothing is wasted after the production of the premium extraction. Key dictates of hashish production have been previously espoused by Clarke [11]: (1) hashish is for devotees with ample supplies of flowers, and (2) the finest quality is obtained by employing purest forms from the start. The approaches outlined herein seem to fulfil these criteria.

Dry ice has been previously employed for cannabis product preparation, but most often it is employed in conjunction with cannabis waste products, such as fan leaves and “shake”, and it is usually employed in direct content with plant material as an abrasive agent that not only dislodges trichomes but also leaf and non-glandular trichome detritus. This is in contrast to the use herein of fresh inflorescence exposed solely to CO_2_ vapor. Dry ice treatment does not require equipment under high pressure, as in supercritical CO_2_ extraction, does not require residual solvents with possible toxicity (e.g., butane) and can be stored in insulated containers for utilization in subsequent runs.

Certain statements on dry ice safety measures are necessary. Firstly, the dry ice employed must be of pharmaceutical grade and devoid of contaminants. The material employed herein was food grade dry ice but was for experimental purposes and not for human use. Secondly, dry ice is extremely cold and will produce severe freeze injuries to exposed skin unless proper gloves and other protective equipment are utilized. Finally, CO_2_ vapor from dry ice is heavier than air and is a toxicant that can accumulate in an enclosed area with a risk of hypoxia, asphyxia and cardiac dysrhythmias unless the workspace is properly ventilated [19,20]. 

A limitation of this study was that single trials and comparisons were carried out on each of the four chemovars without repetition in order to ensure reproducibility. It is certainly possible that different dry ice exposure times or sifting duration may produce optimized extraction results. Future studies with comparison procedure runs may yield additional data that increase the efficiency and yields of the current methods employed.

## 4. Materials and Methods

### 4.1. Production of Cannabis Raw Material

The cannabis chemovars utilized in this study are derived from Prūf Cultivar, a licensed tier 2 indoor controlled environment agriculture (CEA) cannabis producer located in northeast Portland, OR, USA (https://www.prufcultivar.com, accessed on 22 August 2021). This is an independently Clean Green certified facility (https://cleangreencertified.com, accessed on 22 August 2021, a substitute method for organic certification that is currently not allowed by the US Department of Agriculture). In addition to the adult use premium flower market in OR, this company is focused on precision agricultural methods and consistent/diverse chemovar expression. There is a 6800 ft^2^ (632 m^2^) blooming canopy, divided into eight 1000 ft^2^ (93 m^2^) powder-coated steel pods.

### 4.2. Plant Material 

The following four cannabis chemovars were tested in the study:

1. Doug Fir
  Bred by Jeremy Plumb;
  Parentage: Dogwalker (female, F) (bred by OneEye, Portland) × Strawberry Malawi (male, M) (bred by Equilibrium Genetics, Santa Cruz);
  Type I, high THC (25–29%);
  Total terpene content by dry weight up to 6%, alpha-pinene and beta-pinene dominant;
  Nine weeks to maturity;
  Notes: lanky plant with large biomass yields and intense earthy aroma. Strong notes of fuel, possibly rich in thiol/thiolates;
  Hash yield expected: Low;
2. Astral Works
  Bred by Jeremy Plumb;
  Parentage: Harley Tsu (F) (Ringo/SoHum) × Tangerine Haze (M) (Root Cellar)
  Type II, mixed ratio of CBD/THC;
  Total terpene content by dry weight up to 4%. Myrcene or terpinolene dominant;
  Nine weeks to maturity;
  Notes: Lanky plants with massive yields. Inflorescence feature high levels of anthocyanin and powerful floral aroma;
  Hash yield expected: Low;
3. Tangie Biscotti
  Bred by unknown;
  Parentage: unknown;
  Type I, high THC (25–29%);
  Total terpene content by dry weight up to 4%. Myrcene dominant; linalool secondary;
  Nine weeks to maturity;
  Notes: Strong citrus and sweet bread aroma. Winner of multiple awards;
  Hash yield expected: Low to medium;
4. Ursa Major, aka Krypto Chronic
  Bred by Compound Genetics;
  Cereal Killer × Jet Fuel Gelato;
  Type I, high THC (30%);
  Total terpene content up to 5%. Beta-caryophyllene dominant;
  Eight weeks to maturity;
  Notes: a mild aroma on a high THC *wunderkind*, popular in the Oregon market;
  Hash yield expected: Medium to high.

### 4.3. Cultivation Practices

Prūf Cultivar is passionate about organic culture, utilizing highly enriched third-party certified media (living soil) as the foundation of its program. The base organic soil employed (KIS Commercial Mix, Keep It Simple Organics, https://www.kisorganics.com/, accessed on 22 August 2021) consists of biochar, sphagnum peat moss, old mountain fish compost, earthworm castings, volcanic pumice, glacial rock dust, basalt, Calphos soft rock phosphate, oyster shell powder, alfalfa meal, fishbone meal, crustacean meal, kelp meal, fish meal, feather meal, agricultural lime, mycorrhizae, KIS Microbe Catalyst and beneficial microbes.

Media are routinely tested by saturated paste analysis and soil analysis (Logan Labs, Lakeview, OH, USA, https://loganlabs.com, accessed on 22 August 2021). Tissue testing and occasional sap analysis (Apical Crop Science, Canby, OR, USA, http://www.apical-ag.com, accessed on 22 August 2021) are also performed in order to monitor closely sufficiency targets by each essential mineral and to understand transpiration rates and overall nutrition uptake. 

Additionally, feeding is accomplished via a variety of flowable organic inputs by aggressively mixing inputs in a batch mixer and utilizing fertigation with drip stakes. Simple organic amendments, primarily agricultural sulfates, are utilized to achieve overall balance of minerals in the media and plant tissue by applying drenches and foliar applications.

Dissolved oxygen (DO) is enhanced from the ambient 8 parts per million (ppm) to 25 ppm.

Following initial propagation, transplanting is achieved into 5 inch (12.5 cm) vegetative containers and terminates in 10 gallon (37.85 L) individual containers composed of natural fibers and recycled plastic water bottles.

### 4.4. Lighting/Vapor Pressure Deficit (VPD)

The plants used in this trial were all cultivated under fix-mounted light emitting diode (LED) illumination (Fluence Vyper 2, “Physiospec” spectrum, Fluence Science, Austin, TX, USA, https://fluence.science, accessed on 22 August 2021) operated on dimmers. The target photosynthetic photon flux density (PPFD) by the end of vegetative growth is 600, beginning of bloom at 800 and with a peak at 1000 prior to backing off during the last 2 weeks. 

Propagation—100 PPFD/0.8 kilopascal (kPA)

Vegetative Stage 1—200–400 PPFD/1 kPA

Vegetative Stage 2—400–600 PPFD/1 kPA

Flowering Stage 1—600–800 PPFD/1.2–1.5 kPA

Flowering Stage 2—800–1000 PPFD/1.5 kPA

Flowering Stage 3—700–800 PPFD/1.2 kPA

### 4.5. Integrative Pest Management (IPM)

Prūf Cultivar exclusively relies on third party certified inputs and emphasizes biocontrols releasing *Stratiolaelaps scimitus* (formerly *Hypoaspis miles*, predatory mite), *Neoseiulus cucumeris* (thrips predator), *Amblyseius andersoni* and *Amblyseius swirksi* (predatory mites); the occasional use of a sprayable biological anti-fungal (Regalia, Marrone Bio Innovations, Davis, CA, USA, https://marronebio.com/products/regalia/, accessed on 22 August 2021); a broad spectrum algaecide/bactericide/fungicide with hydrogen peroxide and peroxyacetic acid (ZeroTol, BioSafe Systems, Missoula, MT, USA, https://biosafesystems.com/, accessed on 22 August 2021); and organic oils.

### 4.6. Drying and Packing

**Figure 11:**
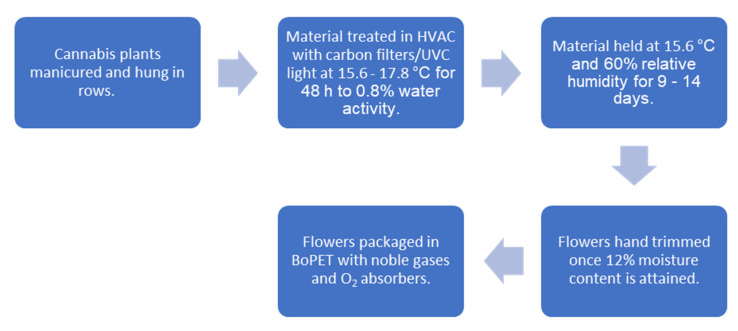
Cannabis drying and packaging schematic.

Harvest involves removing prominent fan leaves and cutting uniform 16–24 in. (41–61 cm) branches and hanging these 2 in. (5 cm) apart on custom vertical trolleys. These carts are loaded into 40 ft. (12.2 m) containers (Conex West, Portland, OR, USA, https://conexwest.com, accessed on 22 August 2021) that are plumbed with laminar flow style HVAC (heating ventilation and air conditioning) units with an initial temperature set point of 60 °F (15.6 °C) with elevations up to 64 °F (17.8 °C) depending on the volume of material and consequent air flow reduction. Intake and outtake ports are distributed in opposition with controlled airflow. The air is scrubbed with carbon filters and ultraviolet C (UVC) light. The initial 48 h requires high levels of dehumidification in order to attain 0.8% water activity to inhibit microbial growth. Once this occurs, the settings are adjusted to 60 °F (15.6 °C) and 60% relative humidity (RH). The duration of drying requires an additional 9–14 days depending on the amount of material and flower size. A moisture analyzer (Sartorius, Göttingen, Germany, https://sartorius.com, accessed on 22 August 2021) is used to validate moisture levels. Once moisture content is reduced to 12%, the flower is carefully hand trimmed and sorted by grade. The trimmed premium flower is finally packed at 10% moisture content in biaxially-oriented polyethylene terephthalate (BoPET) (Mylar, DuPont Teijin Films, Chesterfield County, VA, USA, https://usa.dupontteijinfilms.com, accessed on 22 August 2021) and topped off with a blended noble gas to displace most of the oxygen. Food grade oxygen absorbers are added to 224 g increments of flower before being sealed. Depending on variety and flower morphology, an additional step called “binning” may be applied that allows for “burping” the moisture, ethylene and CO_2_ in a controlled fashion in large plastic containers until the moisture content is 10% before packing. This process is schematized (Figure 11).

### 4.7. Sanitation

Sanitation in cultivation consists exclusively of application of ecological soap and peroxyacetic acid-based sanitizer (PAA) at various concentrations, 0.5–2%. 

### 4.8. Resource Use Efficiency (RUE) Statement

Every new light in the Prūf Cultivar facility or the replacement of existing lights consists of high efficiency LED fixtures. The HVAC is capable of “free-cooling”, which can use scrubbed outside air for air exchanges when ambient temps are lower outside, than in reducing dependency on the chilling plant. Vertical farming is practiced in multiple chambers, with reduction in the waste stream at every turn, most recently with pots composed of a mix of biodegradable fibers and recycled water bottles. The facility produces extremely low run-off outside of cleaning with management of the living soil program without ever requiring watering producing leachate of excess minerals. Plans are for all condensates to be redirected into irrigation. Used media are donated to support organic farms. Prūf Cultivar released our first fully compostable, still compliant packaging for pre-roll products and continues to work to reduce the energy footprint, hoping eventually to support and inspire the development of fully off-grid plant factories. Such facilities could reliably produce food and medicine year-round in a cost-effective, ecological fashion using up to 95% less water and 300 times less farmland than conventional agriculture without pesticides, herbicides or fungicides and with no run-off to adversely affect fresh water sources.

### 4.9. Experimental Procedures for Dry Ice Kief Production

**Figure 12:**
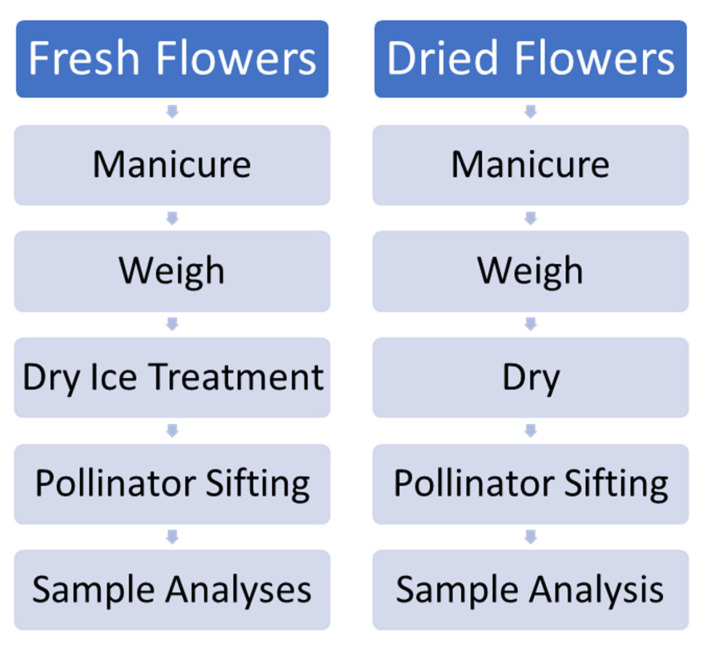
Schematic of procedures employed for experimental fresh and dried flower processing.

Four total trials were undertaken, including two pilot trials with chemovars Doug Fir and Astral Works. The experimental scheme is outlined in Figure 12. Due to an impending snowstorm, a truncated effort with only one hour of dry ice exposure and 5 min of Pollinator treatment was performed with 100 g of fresh material. Subsequent trials with chemovars Tangie Biscotti and Ursa Major employed refined techniques, with 200 g of fresh material rather than 100, and with 48 h of dry ice treatment and 20 min Pollinator sifting treatment.

In each trial, cannabis inflorescences were freshly harvested and quickly manicured by hand in order to remove stems and “sugar leaves” and to separate larger flowers into pieces. Starting weights were carefully measured. Half of the samples were dried and cured as per the above procedure, while the other half was treated by placement in a metal casserole dish on a bed of food grade dry ice pellets (Oxarc, Gresham, OR, USA, www.oxarc.com, accessed on 22 August 2021) within a polyethylene cooler (Gott 48 Quart/45.4 L) and a metal tray placed above with an additional bed of dry ice. The cooler’s drain plug was opened to allow full egress of CO_2_ and water vapor and allowed laminar flow of vapor over and through cannabis inflorescences in order to maximize penetration and lyophilization effects.

After dry ice vapor exposure (1 h vs. 48 h), the cannabis flower material was quickly reweighed and placed within the drum of a sieving cylinder device (150 g model Pollinator^®^, Pollinator Company, Amsterdam, the Netherlands, https://pollinator.nl, accessed on 22 August 2021). This contained a spinning cylindrical drum with 150 µ perforations to allow egress of the largest capitate glandular trichomes.

The Pollinator unit was placed inside a chest freezer (0 °F/−18 °C), and the drum spun at 33 revolutions per minute (RPM, 5 min. for pilot samples, 20 min for subsequent trials). After treatment, inflorescences were reweighed, and trichome material (Kryo-Kief™) were collected for analysis. Two Pollinator drums were employed and cleaned between used with brushing and ethanol treatment.

Equivalent starting weights of each chemovar were dried to an approximate 10% moisture content (vide supra, Section 4.6) and run through the Pollinator machine analogously to the fresh/frozen materials.

**Figure 13:**
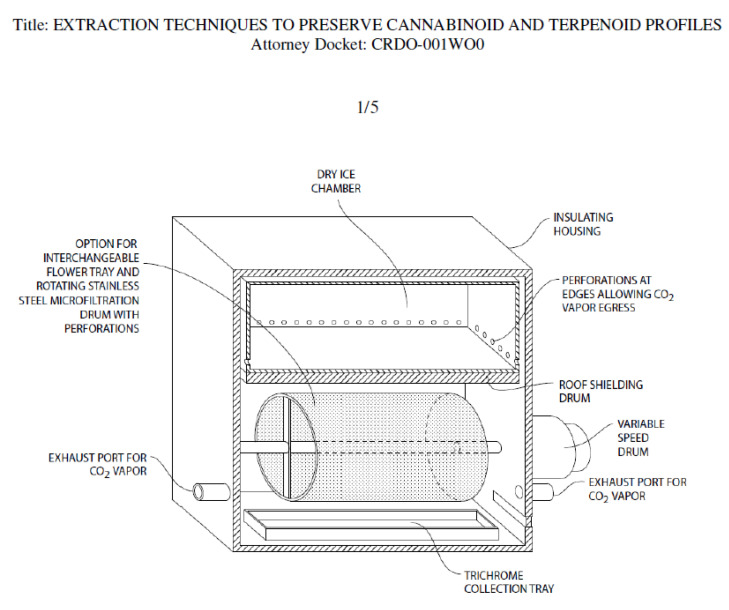
Prototype unit for Kryo-Kief™ extraction (Patent Pending, © 2021 Ethan Russo and CReDO Science).

A drawing of a prototype device for dry ice kief extraction has been filed with the US Patent Office (Figure 13). The design separates fresh cannabis flowers from direct contact with food grade dry ice, while allowing exposure to its vapor.

### 4.10. Sample Analysis

All samples were sent for cannabinoid and terpenoid analysis. This was undertaken at Lightscale Labs, Portland, OR, USA, https://lightscale.com/, accessed on 22 August 2021.

For flower cannabinoid analysis, samples were homogenized using a spice grinder or Magic Bullet blender (depending on the total sample mass), weighing out 100 mg into a Falcon tube, adding 5 mL of high-performance liquid chromatography high performance liquid chromatography (HPLC)-grade methanol, vortexed for 15 min, centrifuged at 3000 RPM for 10 min and finally diluted 50 µL of the extract into 950 µL of HPLC-grade methanol for a 1:20 dilution (100-fold dilution factor). 

For extract cannabinoid analysis, the samples were homogenized using a stir rod, weighed out 50 mg into a Falcon tube, 5 mL of HPLC-grade methanol, vortexed for 15 min, centrifuged at 3000 RPM for 10 min and finally diluted 50 µL of the extract into 950 µL of HPLC-grade methanol for a 1:20 dilution (100-fold dilution factor). The samples were analyzed via a 1 μL injection onto a column (Restek Raptor ARC-18 150 mm × 4.6 mm (2.7 μm), Restek Pure Chromatography, Centre County, PA, USA, https://www.restek.com, accessed on 22 August 2021) maintained at 50 °C with a binary mobile phase (Mobile Phase A: 0.1% formic acid in LCMS-grade Water; and Mobile Phase B: 0.1% formic acid in HPLC-grade acetonitrile) gradient at 1.5 mL min^−1^ consisting of a time program of *t* = 0 to 4 min (70% A: 30% B to 100% A: 0% B) and *t* = 4.1 min to 7 min (70% A: 30% B). Multi-wavelength detection (major cannabinoids THCA: 275 nm; CBDA: 268 nm; THC: 228 nm; and CBD: 228 nm) on an HPLC (Nexera-i LC-2040C 3D, Shimadzu, Santa Clara, CA, USA, https://www.shimadzu.com, accessed on 22 August 2021) equipped with a photodiode-array detector (PDA) allowed ultraviolet-visible spectroscopy (UV-Vis) confirmation in conjunction with calibrated retention times (detection bandwidth = 0.05 min) of 11 cannabinoids. The HPLC was linearly calibrated for quantification from 1 ppm to 800 ppm by using certified reference materials (Cayman Chemical, https://www.caymanchem.com, accessed on 22 August 2021).

For flower and extract terpenoid analysis, 20 mg of samples was weighed out from the homogenized sample into a gas chromatography (GC) headspace vial and capped/crimped to hermetically seal the vial. The terpenoid analysis employed a gas chromatography-mass spectrometry full evaporation technique (GCMS FET) method with a GCMS (GCMS-QP2010 S, Shimadzu, vide supra) coupled to a HS-20 headspace sampler. The samples were allowed to equilibrate in the HS-20 at 140 °C for 10 min prior to injection into the transfer line. The samples were analyzed via a 1 μL split injection (split ratio = 1.0) onto a Rxi-624Sil MS 30 m × 0.25 mm (inner diameter) and 1.4 μm thickness (Restek, vide supra) with ultrahigh purity helium (carrier gas) at 0.83 mL min^−1^ (46.3 kPa). The GC oven was ramped from 60 °C to 300 °C at 12.5 °C min^−1^ with a final bakeout at 300 °C for 2.5 min for a total cycle time of 28 min. Terpenoid detection occurred with an interface temperature and ion source temperature of 310 °C and 250 °C, respectively. Scan mode from 35 to 250 *m*/*z* (independent variable in a mass spectrum) with a scan speed of 2500 was utilized from 6 to 18 min during the GC cycle time for detection. The mass spectra of identified terpenoids were cross-referenced relative to the 2014 National Institute of Standards and Technology (NIST) library and with a >90% confidence level. With calibrated retention times, the GCMS was quadratically or linearly calibrated (depending on terpenoid response) from 10 ppm to 2500 ppm using Cayman Chemical certified reference materials.

Analytical results were tabulated to allow comparison of each of the six different conditions: fresh flower, frozen-sifted flower by-product, Kryo-Kief™ (dry ice kief), dried flower, dried sifted flower and dried kief (vide supra, Results).

## 5. Conclusions

This article introduces a novel solventless extraction technique for cannabis that utilizes fresh frozen flowers as a feedstock for high potency, high purity cannabis trichome extracts without extraneous materials. Dry ice extraction of fresh flower preserves monoterpenoids in a manner that may prove more therapeutic in clinical practice. The combination of alternative secondary processing techniques after dry ice kief isolation of trichomes portends to provide phytocannabinoid and cannabis terpenoid fractions of the highest quality and purity, with little to no chlorophyll or extraneous lipid components. The concentrations achieved with this technique rival those for cannabinoid content from solvent extractions and exceed them in terms of terpenoids.

Observed yields in the latter experiments are quite competitive with other extractions, e.g., “water hash” [11]. The remaining material after Kryo-Kief™ processing, which is the frozen-sifted flower by-product, is not wasted but can undergo secondary processing by cold ethanol or supercritical CO_2_ extraction for various markets and purposes.

The above approaches can be combined with other techniques (e.g., nanofiltration and centrifugal chromatography) to produce any desired combination of acid and neutral phytocannabinoids and cannabis terpenoids that may be tailored to specific therapeutic indications or industrial applications.

As exemplified in Methods, such techniques offer the capability of reproducible production of pure cannabinoid and terpenoid extracts or isolates from scrupulously organic cultural practices that will be suitable for application in pharmaceutical or cannabis supplement development programs. Such approaches may simplify resulting extracts and their biochemical fingerprints in a manner that may enhance standardization and facilitate regulatory approval through Chemistry, Manufacturing and Control (CMC) [21].

## 6. Patents

Ethan Russo has filed a patent application with the US Patent Office, CRDO-001WO0, on the extraction technology of Kryo-Kief™ described in this article.

## Data Availability

Data supporting reported results are held by E.B.R.

## Sample Availability

Samples of the materials are not available from the authors due to legal constraints.
